# Does Trans-radial Longitudinal Compression Influence Myoelectric Control?

**DOI:** 10.33137/cpoj.v5i2.37963

**Published:** 2022-07-20

**Authors:** J Olsen, S Day, S Dupan, K Nazarpour, M Dyson

**Affiliations:** 1 Intelligent Sensing Laboratory, School of Engineering, Newcastle University, UK.; 2 National Centre for Prosthetics and Orthotics, Strathclyde University, UK.; 3 Edinburgh Neuroprosthetics Laboratory, School of Informatics, The University of Edinburgh, UK.

**Keywords:** Amputation, Prosthetic, Socket, Compression, Myoelectric, EMG, Control, Fatigue, Compression-Release, Trans-Radial, Upper-Limb

## Abstract

**BACKGROUND:**

Existing trans-radial prosthetic socket designs are not optimised to facilitate reliable myoelectric control. Many socket designs pre-date the introduction of myoelectric devices. However, socket designs featuring improved biomechanical stability, notably longitudinal compression sockets, have emerged in more recent years. Neither the subsequent effects, if any, of stabilising the limb on myoelectric control nor in which arrangement to apply the compression have been reported.

**METHODOLOGY:**

Twelve able-bodied participants completed two tasks whilst wearing a longitudinal compression socket simulator in three different configurations: 1) compressed, where the compression strut was placed on top of the muscle of interest, 2) relief, where the compression struts were placed either side of the muscle being recorded and 3) uncompressed, with no external compression. The tasks were 1) a single-channel myoelectric target tracking exercise, followed by 2), a high-intensity grasping task. The wearers’ accuracy during the tracking task, the pressure at opposing sides of the simulator during contractions and the rate at which the limb fatigued were observed.

**FINDINGS:**

No significant difference between the tracking-task accuracy scores or rate of fatigue was observed for the different compression configurations. Pressure recordings from the compressed configuration showed that pressure was maintained at opposing sides of the simulator during muscle contractions.

**CONCLUSION:**

Longitudinal compression does not inhibit single-channel EMG control, nor improve fatigue performance. Longitudinal compression sockets have the potential to improve the reliability of multi-channel EMG control due to the maintenance of pressure during muscle contractions.

## INTRODUCTION

Modern trans-radial limb prostheses comprise three main elements: a state-of-the-art bionic hand,^1,2^ sensors for capturing electromyographic (EMG) signals, and a socket - the design of which has not changed significantly in over 60 years.^3–5^

The introduction of the Muenster and Northwestern style sockets led to the emergence of self-suspending trans-radial prostheses as early as 1960s.^4,6,7^ These designs eliminated the need for a suspension harness,^7^ giving more freedom to wearers.^8^ Around a decade later, EMG-controlled terminal devices became prevalent. The EMG sensors, which are required for control, were retrofitted into self-suspending socket designs.^3^ Since then, there has been a vast increase in the complexity of myoelectric devices available.^9^ Despite this, trends indicate that abandonment rates have not reduced over time, with reports as high as 44% in literature.^10^ Lack of control, poor reliability and discomfort are key causes of abandonment of myoelectric prostheses.^5,11–16^

Traditional socket designs are not optimised to accommodate the weight of additional hardware or to prevent loss of contact between the EMG sensors and their target muscle groups.^3^ Restricted space within most sockets generally only allows for one or two clinical-standard electrodes.^17^ Additionally, some modern terminal devices exceed 0.6kg,^18^ approximately three times the weight of a split-hook, a common body-powered alternative.^19^ Adjustable electrode housings have been trialled in an attempt to assist myoelectric control with existing sockets.^15^ However, there are no known novel socket styles designed specifically to optimise EMG control, and research into this topic is scarce.^16^ In contrast, several designs have emerged with the aim of improving biomechanical stability, most notably those featuring longitudinal compression.^7,20–22^ It is known that consistent contact between the residuum and the electrodes is required for reliable myoelectric control,^3,16^ but to the best of our knowledge there is currently no published research detailing whether the enhanced tissue stabilisation provided by longitudinal compression sockets improves myoelectric prosthesis reliability. Out of the available longitudinal compression socket designs, the Compression-Release Stabilized (CRS) socket is a well-known design for which fitting notes are documented.^20^

The theory behind longitudinal compression sockets is that the compressed areas stabilise the underlying structures and reduce lost-motion, the relative motion between a socket and residuum during movement, improving biomechanical stability.^20^ Relatively recent designs, such as the CRS^20^ feature both longitudinal compression and cutout release regions for the displaced tissue to spill into.^20,21^ Earlier iterations of sockets featuring localised compression such as the “*Trans-radial Anatomically Contoured (TRAC) interface*”^7^ and the “*Anatomically Contoured and Controlled Interface (ACCI)”*^22^ did not feature release areas to allow the displaced tissue to move into, and therefore had limited success. This paper will therefore reference the CRS design to explain the fundamental principles of longitudinal compression sockets. Note that throughout the paper we have referred to longitudinal compression as a concept, not a specific socket design.

Conventional CRS sockets are fitted using a protected procedure which only trained professionals can perform.^20^ The process involves bar-shaped depressors indenting the residuum during the casting stage to create areas of intentional localised compression.^20^ The location of the bars is determined by the professional conducting the CRS cast, based on underlying tissue geometry and avoiding major blood vessels.^20^ Currently there is no public guidance or published scientific evidence to suggest which sensor location in a CRS socket is more beneficial for myoelectric control. In the original paper that proposed the CRS design,^20^ the image of the socket are contradictory. The image shows the electrodes mounted on compression struts, but the text suggests that they could be placed on a membrane in the relief area. Anecdotally, it is known that in sockets featuring depression bars, such as the CRS, electrodes are usually mounted in compressed areas for convenience and several images of CRS sockets support this.^20,23^

Other positive effects that longitudinal compression sockets may have on residuum physiology are yet to be reported. Compression garments are frequently used therapeutically for medical conditions such as oedema and cerebral palsy and to improve athletic performance.^24–30^ As longitudinal compression sockets provide regions of both high and low pressure, it is assumed their mechanism of action will be similar to that of “*directional compression*” garments, which provide targeted areas of varying compression.^26^ Directional compression garments have been shown to reduce physiological responses which would result in muscle fatigue during sport and physical activity,^27,28^ however it is not yet known whether longitudinal compression sockets provide the same benefit. Additionally, high pressure must be applied with caution, as excessive localised compression can result in tissue ischemia and skin breakdown.^29,31^ If the pressure restricts blood flow for a significant period of time, wounds, injuries and even tissue death can occur.^20,31,32–34^

Finding an acceptable level of compression and blood perfusion is a complex task for prosthetists without additional equipment.^20^ No quantitative method or guidelines are available, however postischaemic hyperemia (redness after a prosthesis is removed) can be used to gauge acceptable compression levels.^20^ Extrapolating existing data for medical devices is also complex as many studies reporting safe levels of compression for medical devices refer to stockings which provide a different mechanism of compression.^33^ Additionally, the safe range for compression garments depend on the location compression is being applied to.^29,31,32–35^ Similarly, studies of localised pressure often refer to pressure sores resulting from long-term tissue ischaemia in immobile patients.^29,35^

This study explored the potential effect of longitudinal compression on three fundamental factors central to the use of myoelectric prostheses; namely, control, electrode-skin contact and muscle fatigue. We hypothesised longitudinal compression would provide enhanced myoelectric control due to immobilisation of the target muscles.

## METHODOLOGY

The local ethics committee at the Newcastle University approved this study (Ref: #11532/2020 and #20-DYS-050). Twelve able-bodied participants between 20-40 years of age were recruited (sex: 7 male, 5 female). All participants were active individuals who self-identified as right-hand dominant. As our participant pool was limited in size, and we did not anticipate factors such as mass, height, or grip strength to be associated with myoelectric ability; only participant gender and age range were recorded.

A two-part experiment featuring a custom-made longitudinal compression simulator was performed. The first part of the experiment assessed the effect of longitudinal compression on EMG control using a simple target tracking task. The second part assessed the effect of longitudinal compression on the rate of forearm fatigue during a short, high intensity grasping activity.

### Equipment

**Figure 1:**
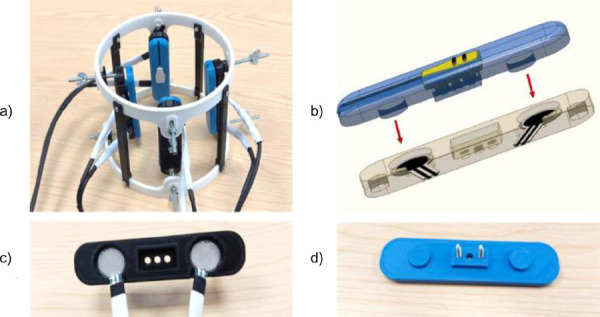
(a): The 3D-printed compression rig; (b): A CAD representation of the compression bar showing the inbuilt FSR depressors; (c): The top half of a compression bar, showing the FSR sensors inside; (d): The bottom half of a compression bar, showing the FSR depressors.

To enable longitudinal, localised forearm compression, a custom rig was developed, shown in **Figure 1(a)**. The rig had four depressor bars, simulating the struts of a longitudinal compression socket. This design was chosen as it is reported to be the most stable configuration for a CRS socket,^20^ a common and well documented example of a longitudinal compression socket. The bars were evenly spaced around the rig. Each bar contained two Ohmite FSR07CE Force Sensing Resistors (FSRs) to allow the compression applied to be calibrated and monitored. Bars could be depressed and released using manually adjustable wing-nuts to fit all participants. Each bar was 3D printed in two halves featuring recessed areas to house the FSRs and depressors to evenly compress the FSRs, as shown in **Figure 1**. The inner-design of the depressor bars allowed reliable calibration of the FSRs prior to use due to the rigid material and consistent depressor area, as shown in **Figure 1(b)**. Each FSR was calibrated between 0-20kPa (≈ 0-150mmHg) using calibration weights. During both calibration and the experiment, pressure data was recorded using a Teensy^®^ 4.0 board. The Teensy ran Firmata firmware and sampled pressure data at 1000 Hz. EMG sensors (DELSYS Mini, DELSYS, USA) were used to acquire EMG data at 2000 Hz. The AxoPy experimental library was used to synchronize pressure and EMG data, and to provide online visualisation.^36^ Two dynamometers (CAMRY, USA) were used during the fatigue experiment.

#### Safety

Given that there was no documented precedent for the appropriate level of compression to apply, it was calculated based on the task duration. Chang et. al established a parabolic relationship between the length of time that tissue is compressed, magnitude of compression, and safety.^32^ Assuming no shear forces, the relationship is valid for between 2 to 7 hours of compression. The task was predicted to take 2 hours approximately, hence the maximum safe pressure level was calculated to be 16kPa (120mmHg). To ensure safety and make the results more applicable to daily wear of a myoelectric prostheses, the target range of compression was lowered to 6.7-9.3kPa (50-70mmHg), which would give an approximate allowable wear time of 3.4-4.8 hours, with a tolerance range of 5.3-10.7kPa (40-80 mmHg) per bar. It is important to note that although no numerical precedent is documented, the CRS socket “*compress the tissue against the long bone [...] until it no longer yields*”,^37^ which is much higher than the levels featured in this experiment as even at the upper range of 10.7kPa, the limb were not completely compressed. During calibration a real time display provided a colour coded pressure value data from each FSR to the experimental operator to facilitate calibration.

### Experiment

**Figure 2:**
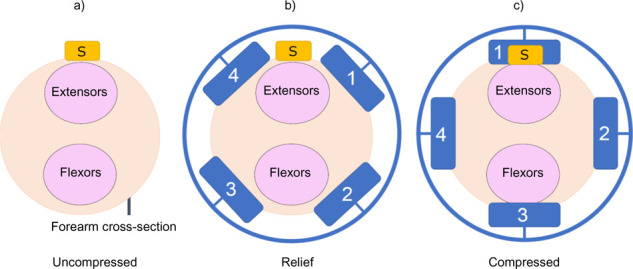
Experimental conditions tested. The approximate location of the wrist extensors and flexors are shown relative to the positions of the rig during the different data acquisition configurations and the corresponding locations of bar 1, 2, 3 and 4. “S” represents the location of the EMG electrode throughout all three configurations. (a): Uncompressed configuration; (b): Relief configuration; (c): Compressed configuration.

Three compression-release socket configurations were tested. Each condition changed the location of the compression bars while an EMG sensor remained fixed in an identical location on the extensor muscle group. The socket configurations tested are shown in **Figure 2** and were defined as follows:

Uncompressed: The EMG sensor was affixed to the skin with no external compression.

Relief: The EMG sensor is located in the relief area, equidistant between two compression bars.

Compressed: The EMG sensor is located underneath a compression bar.

For both the uncompressed and relief configurations, a DELSYS adhesive interface (adhesive film) was used to affix the EMG sensor to the skin. For the compressed configuration this was not required as the compression bar held the sensor in place.

#### Control

**Figure 3:**
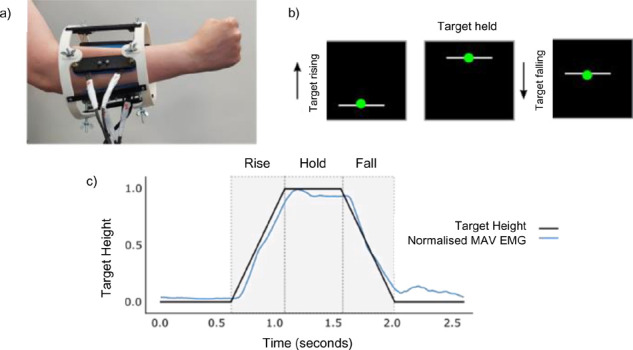
a) A photo of the compression simulator being worn. Note the limb is extended more than the 90° than described in the text to obtain a clear picture. b) An example of what the task looked like on screen as presented to the participants. The white line is the moving target, and the green ball is the cursor which participants control with their EMG activity. c) An example plots from a high-target task with the corresponding EMG activity showing the participant tracking the height of the cursor. Only the three gray areas highlighted in the graph were used to calculate participant scores, corresponding to the rise, the hold period, and fall of the on-screen target.

Prior to each experiment a calibration process was performed wearing the simulator as shown in **Figure 3** **(a)**. Participants were asked to position their dominant arm at their side, with 90-degree elbow flexion and their wrist in a neutral position. Participants were shown how to contract their wrist extensors using wrist motions and the extensor muscle group was manually located by palpating the arm. The EMG sensor was placed on the extensor area and the quality of the acquired EMG signal was confirmed by visual inspection. The location of the electrode was then marked using a marker pen.

An EMG calibration procedure was performed.^38^ Holding the aforementioned neutral position, a mean absolute value (MAV) was captured over a 750ms window, representative of two states: baseline EMG activity (y_min_), and a comfortable contraction (y_max_). It was explained that participants would need to repeat this contraction many times throughout the experiment, hence they should not contract too much to prevent future discomfort. The MAV of the raw EMG data input was denoted as (y). Normalisation constants were derived from calibration MAV data, and in all consequent conditions EMG was normalised using said constants. Normalised muscle activity (y_norm_) was calculated as:


(1) $$y_{\text{norm }}=\left(y-y_{\min}\right)/\left(y_{\max}-y_{\min}\right)$$

In all experiments y_norm_ was used for control. Each participant was calibrated in the experimental condition they performed first. For further details of the calibration procedure see the methods described in Dupan et. al.^38^

A simple, 1-dimensional myoelectric target tracking task was used to test control. The task visuals and processing were written in Python, using the AxoPy library.^36^ The task comprised dynamic on-screen targets which rise, hold and fall from the minimum EMG value scaled to two target heights: 25% and 100% of the comfortable EMG contraction, as shown in **Figure 3(b)**. Participants were instructed to hold their arm in the position established during calibration and to track the target with the cursor.

The cursor was controlled by the normalised muscle activity of the extensor group, as shown in **Figure 3(c)**. Each task block consisted of 20 trials - 10 low targets and 10 high targets displayed in random order. Each trial was the same duration, regardless of whether the target was low or high, hence the high targets moved faster than the lower targets to rise, hold and fall within the same timeframe. Participants completed one familiarisation block of 20 trials, which was not included in the analysis. Four blocks of 20 trials were recorded in each configuration producing a total of 240 trials per participant. Each participant performed the control task in all three configurations. The testing order for the configurations was balanced between participants.

Data from each control trial was split into three time-periods: rise, hold, and fall, corresponding to the target motion. The absolute deviation of the normalised MAV from the target was calculated for each data point, and a numerical mean calculated. Participant averages were calculated to provide twelve average scores per time-period, per configuration. Score distributions were checked for normality using a Shapiro-Wilks test. The majority of data sets were found to be non-normally distributed (p < 0.05). Friedman tests were used to check for statistical differences between the three rig configurations for: 1) the rise, hold and fall section of the trial, and 2) between the low and high targets.

#### Pressure

For configurations relief and compression, the pressure applied by the rig was fine-tuned manually before commencing data acquisition. The acceptable pressure range was 5.3-10.7kPa (40-80 mmHg) with the arm in the neutral position, with the ideal range being 6.7-9.3kPa (50-70mmHg). During the compressed configuration, bar 1 compressed the approximate area of the extensors and bar 3 compressed the approximate area of the flexors. Although both were within the target 6.7-9.3kPa (50-70mmHg) pressure range, the pressure exerted onto the extensors by bar 1 was consistently around 2kPa (15mmHg) higher than that exerted onto the flexors by bar 3. This is due to anatomical differences. The extensors are a larger muscle group than the flexors, providing more cushioning and tissue compliance. Additionally, bar 1 is aligned with the belly of the extensors, whereas bar 3 is closer to the bone and above the approximate area of the flexors. The enhanced tissue cushioning and alignment of bar 1 allow a higher pressure to be achieved than bar 3. It is assumed that individuals with acquired limb differences would generally have a similar muscle structure to the able-bodied volunteers, however individuals with congenital limb differences would show more varied limb structures. Regardless, the simulator was designed to be fine-tuned to fit each individual’s limb, with the aim of achieving approximately equal compression provided by all four bars.

The intention of this analysis was to gauge whether longitudinal compression could prevent electrode lift-off. Hence, only the compressed configuration data was assessed for this section as it allows recording of both EMG and pressure data directly above the EMG site. The average rise and fall of pressure recorded from bar 1, the EMG-bearing extensor bar, and bar 3, the flexor bar, throughout all compressed trials was calculated to assess the effect of muscle contraction on EMG sensor pressure within the compression simulator.

Data recorded during compression conditions were separated into two groups: high targets and low targets. For both groups, data points recording pressure change and EMG activity were averaged to observe mean fluctuation during the trial.

#### Fatigue

The effect of longitudinal compression on forearm fatigue was tested using a bi-manual task. Participants’ forearm extensors were located on both arms as described in section *Control* and an EMG sensor was affixed to both forearms above the extensors. The position of the sensors was validated on screen as described in section *Control*. The compression simulator was applied to one arm as described in the compressed configuration. Participants were asked to grip two identical dynamometers, using their maximum grip strength i.e., a sustained isometric maximal contraction, for as long as they felt they could, and to release them simultaneously. This test was based on similar methodology described by Klass et. al^39^ and Gillani et. Al.^40^ Handheld dynamometers were chosen for this experiment to avoid the use of unnecessary custom hardware. Testing order was balanced so that compression was applied to the dominant arm and non-dominant arm on an equal number of instances to minimise the effect of structural differences.^41–44^ The physiological effects of fatigue on muscles vary depending on the intensity and duration of the fatiguing task, as well as the muscle being observed.^24,45^ Pilot experiments were conducted, and the volunteers reported feeling muscle fatigue for several hours after conducting the single maximal grip strength task. Due to this, the fatigue task was only performed once per participant to avoid a multi-day experiment which may have introduced more variance between performance. The two configurations selected to be compared were uncompressed and compressed, as this allowed a direct comparison of the extensors with and without external pressure. Hence, the relief configuration was eliminated for this task.

For each participant’s individual pair of compressed and uncompressed EMG recordings, the “*active data*” was analysed, i.e., the entire duration of the participant’s contraction. The length of each pair of recordings varied depending on how long the participant contracted their muscles during the fatigue task. Hence, for each condition, a median frequency analysis was performed using 1 second intervals. Observing changes to the median frequency of an EMG recording is a well-established method of gauging muscle fatigue.^46^ A percentage difference was calculated for each participant, based on the difference between the first and last datapoints of the median frequency analysis. Shapiro-Wilks tests were used to check for normality in percentage decreases. None of the datasets were found to be non-normally distributed (p < 0.05). Wilcoxon’s rank (p < 0.05) was used to check for significance between the conditions. The Shapiro-Wilks test and Wilcoxon’s rank analysis were repeated with data split into dominant arm recordings and non-dominant arm recordings, to assess whether limb dominance influenced fatigue.

## RESULTS

Experimental results from the control task, the pressure analysis and the fatigue task are detailed in the following sections.

### Control

**Figure 4:**
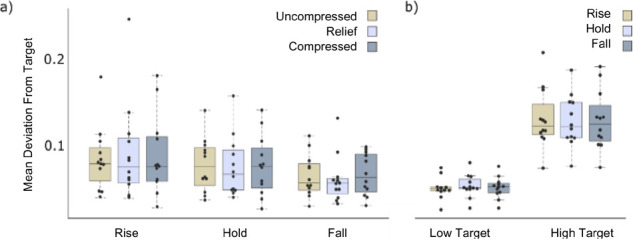
Results from the myoelectric target tracking control tasks. Mean absolute deviation from the target for (a) the rise, hold and fall periods for all trials (b) low targets and high targets. In all box plots, the upper and lower box boundaries represent the respective upper and lower quartiles, the whiskers represent the maximum and minimum excluding outliers, and the centre line represents the median.

Average scores for the rise, hold and fall period of the task are shown in **Figure 4(a)**. Average scores for low target and high target trials for each condition are shown in **Figure 4(b)**. There was no significant difference between any conditions during the rise (p = 0.717), hold (p = 0.920) and fall (p = 0.717) periods. The results for the rise, hold and fall periods were similar, with a small decrease in error for the fall period. As would be expected, there was a notably higher error for the faster-moving high target trials than low target trials. However, there was no significant difference (p < 0.05) in average scores between conditions for either high (p = 0.77) or low (p = 0.368) targets. An assessment of individual participant performance revealed a weak trend R^2^ = 0.349 of error reduction as the trials progressed, shown in **Appendix A**.

### Pressure

**Figure 5:**
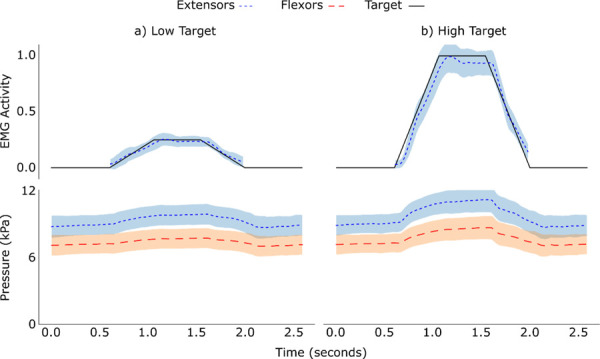
The mean EMG recording and corresponding pressure recordings from the extensors (shown in blue) and flexors (shown in red) from a) the low target trials and b) the high target trials, across all compressed trials from all participants. The black line represents the EMG target height, and the shaded bands show the standard deviation. For the EMG recordings, only the period where the target is rising, held, or falling in height is shown, as participants where not assessed outside of this period. The pressure recorded above the extensors was consistently around 2kPa (15 mmHg) higher than the pressure recorded above the flexors due to anatomical differences described in detail in section 2.2.2.

**Figure 5(a)** shows the mean fluctuations in pressure data recorded during all trials split by high and low targets for bar 1, located above the wrist extensors, and bar 3, located approximately above the wrist flexors, and **Figure 5(b)** shows the corresponding EMG data. Recordings from both the extensor bar and flexor bar showed an increase in pressure during contractions at the opposing sides of the rig for both high and low targets. Due to the anatomical differences described in section *Pressure* (Methodology), the pressure recorded from bar 1, above the extensors, was consistently around 2kPa (15 mmHg) higher than the pressure recorded from bar 3, above the flexors. Despite this, the fluctuation followed the same pattern for both bars in both high and low target groups.

The results of this test showed that pressure rose at opposing sides of the socket simulator during contractions.

### Fatigue

**Figure 6:**
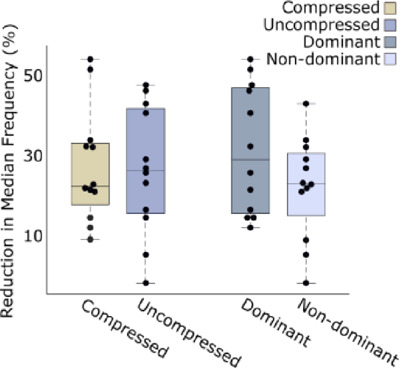
Rates of fatigue for the dominant vs. nondominant arm, and the compressed vs. uncompressed arm. The rate of fatigue is measured as the scalar of the trendline for the median frequency analysis of EMG recordings of each arm. The upper and lower box boundaries represent the respective upper and lower quartiles, the whiskers represent the maximum and minimum excluding outliers, and the centre line represents the median.

**Figure 6** shows a comparison of rates of fatigue for the dominant vs. non-dominant arm, and the compressed vs. uncompressed arm. There was no significant difference in the mean rate of fatigue between participants’ arms in the compressed and uncompressed conditions (p = 0.182), but the mean reduction in median frequency was marginally lower for the compressed configuration than the uncompressed. Similarly, there was no significant difference between the dominant and nondominant arm rates of fatigue (p = 1). The results of this test showed that longitudinal compression applied to the forearm muscles during a high-intensity task did not produce the fatigue-reducing effect observed with compression garments.^28^

## DISCUSSION

The aim of this study was to assess and quantify the effect of longitudinal compression on fundamental factors affecting EMG prosthesis wearability: control, maintenance of contact between the electrodes and the skin, and fatigue.

The results of this investigation indicated that moderate longitudinal compression had no significant effect on the participants closed-loop control abilities in our myoelectric target tracking task. On average, the participants showed a weak trend of improvement (R^2^ = 0.349) as the control task progressed, as shown in **Appendix A**. This trend is likely to be indicative of participants learning to perform the task and will account for some of the variability within the scores. Given the data presented it is unlikely that this variability influenced the results. The results from the control task indicate that when selecting a socket design featuring selective longitudinal compression, alternative factors such as fit and comfort should be prioritised over the EMG control capability provided by the socket.

Most conventional clinical trans-radial sockets feature a rigid socket design within which EMG sensors are recessed into the socket wall.^3^ The extensor carpi radialis and flexor carpi radialis are common muscle sites for dual-channel EMG control, located approximately equidistant around the forearm. This design is susceptible to “electrode lift-off” - during movements, contractions or loadbearing, the residual limb presses against one side of the socket.^3,15^ This can cause the opposing side to disengage with the socket wall and the electrode embedded within it, leading to a loss of contact between the electrode and skin.^3,15^ Pressure data recordings during compressed configuration trials, as shown in **Figure 5**, suggest that integrating electrodes into longitudinal compression bars can be used to maintain pressure at the socket-skin interface during muscle contractions. This study used a simulator as using real sockets was out of scope for the research. Hence, a follow-on study utilising real sockets should be conducted.

Rates of forearm fatigue observed during a short burst of intense physical activity did not differ between compressed and uncompressed arm conditions, however the reduction in median frequency was marginally smaller for the compressed configuration, i.e., the limb fatigued slightly less than in the uncompressed configuration. No significant difference was observed in rates of fatigue between the dominant and non-dominant limb, making it unlikely that this balancing condition had any influence on results. It is important to note that, due to the lack of specialised equipment, this study featured a standard dynamometer and tested hand-grip strength rather than fatiguing the wrist extensors. Commonly, studies assessing compression for sporting purposes are conducted over several, longer recording sessions,^24,26,27,47,48^ whereas this study looked at one recording of maximum muscle contraction from the participants. Further research is therefore necessary to be certain about any relationship between longitudinal compression and limb fatigue.

In summary, both the myoelectric control and fatigue data indicated that the properties of longitudinal compression sockets have little influence on factors relevant for EMG based control of an upper-limb prosthesis while pressure data suggests longitudinal compression bars could be used to maintain electrode contact during prosthesis use. Compression struts in longitudinal compression sockets are intended to displace tissue in order to reduce lost motion. Further research will be necessary to determine whether it is possible to design struts which are able to displace tissue whilst also sensing the EMG activity at a signal to noise ratio sufficient for prosthesis control.

Able-bodied participants were recruited to minimise the effect of variation in limb length and structure. This allowed a fair comparison between different compression configurations. Hence, a simulator was designed to allow the inclusion of able-bodied volunteers. The literature linking compression simulators to real longitudinal compression sockets is sparse, with the only known previous example being Sang, et al.^49^ It is assumed that the majority of acquired trans-radial amputees would have a similar muscle structure to able-bodied individuals, however they may require shorter or narrower compression bars, to suit the length and shape of their residuum. Future experiments should include amputees, ideally those who regularly use a myoelectric device.

### Limitations

As preliminary research in this area, this study featured a number of limitations. The socket simulator designed for this study did not allow for any form of distal loading to simulate wearing a terminal device. Loading will affect many of the factors analysed in this study and will be considered in follow-on studies. Additionally, the control task and pressure data were captured at 90 degrees elbow flexion only. To further understand the effect of longitudinal compression on myoelectric control, future experiments should capture a variety of arm positions. This socket simulator also featured compression bars in an equidistant design around the limb. This design allowed us to test whether localised, longitudinal compression altered EMG properties for single channel control. Adjustable compression bar positions will be necessary to test whether results generalise to multi-channel EMG and pressure-maintenance across various sensor sites.

## CONCLUSION

Longitudinal compression in an equally distributed 4-bar socket simulator does not inhibit single-channel EMG control, nor does it improve fatigue performance of the wrist-extensors during a high-intensity, short-duration contraction. Pressure data reported in this study indicated that longitudinal compression, when applied tangential to the muscle, help maintain overall contact between the skin and the socket at opposing sides. Therefore, longitudinal compression sockets may improve multi-channel EMG control in a design which integrates the EMG sensors into the compression struts.

## DECLARATION OF CONFLICTING INTERESTS

The authors declare that the research was conducted in the absence of any commercial or financial relationships that could be construed as a potential conflict of interest.

## AUTHOR CONTRIBUTION

**Jennifer Olsen**: writing (original draft preparation).

**Jennifer Olsen, Sarah Day, Sigrid Dupan, Kianoush Nazarpour, Matthew Dyson**: conceptualization, writing (review and editing).

All authors have read and agreed to the published version of the manuscript.

## SOURCES OF SUPPORT

This work was supported by the Engineering and Physical Sciences Research Council (EPSRC), U.K., under studentship number 2281137 from EP/N509528/1 and EP/R51309X/1 (JO).

## ETHICAL APPROVAL

The local ethics committee at Newcastle University approved this study (Ref: #11532/2020 and #20-DYS-050).

## Appendix A:

All participants’ mean average deviation from target over trials
